# Pseudo-plaque reduction neutralization test (PPRNT) for the measurement of neutralizing antibodies to Crimean-Congo hemorrhagic fever virus

**DOI:** 10.1186/1743-422X-10-6

**Published:** 2013-01-03

**Authors:** Nurettin Canakoglu, Engin Berber, Mustafa Ertek, Mustafa D Yoruk, Sukru Tonbak, Yusuf Bolat, Munir Aktas, Ahmet Kalkan, Aykut Ozdarendeli

**Affiliations:** 1 Department of Virology, College of Veterinary Medicine, Firat University, Elazig, Turkey; 2 Refik Saydam National Public Health Agency, Ankara, Turkey; 3 Department of Parasitology, College of Veterinary Medicine, Firat University, Elazig, Turkey; 4 Department of Infectious Diseases and Clinical Microbiology, Medical Faculty, Karadeniz Technical University, Trabzon, Turkey; 5 Department of Microbiology, School of Medicine, Erciyes University, Kayseri, Turkey

**Keywords:** CCHF, CCHF-neutralizing antibodies, Pseudo-plaque reduction neutralization test, Fluorescent focus reduction neutralization test

## Abstract

**Background:**

Crimean-Congo hemorrhagic fever virus (CCHFV) is a tick-borne virus of the genus *Nairovirus* family Bunyaviridae, which are enveloped viruses containing tripartite, negative polarity, single-stranded RNA. CCHF is characterized by high case mortality, occurring in Asia, Africa, the Middle East and Europe. Currently, there are no specific treatments or licensed vaccines available for CCHFV. Recently, two research groups have found adult mice with defective interferon responses allowed to lethal CCHFV infection. These mouse models could provide invaluable information for further studies. Efforts to develop a vaccine against CCHFV are being made. To determine the efficacy of vaccine candidates it is important to conduct serological studies that can accurately measure levels of protective antibodies. In the present study, a pseudo-plaque reduction neutralization test (PPRNT) based on enzyme-catalyzed color development of infected cells probed with anti-CCHFV antibodies was used to measure neutralization antibody of CCHFV.

**Methods:**

Sixty-nine human serum samples (20 acute and 49 convalescent) were tested. The presence of CCHFV antibodies was determined and confirmed by a commercial ELISA kit. CCHFV RNA was determined by RT-PCR. All the samples were analyzed by PPRNT and fluorescent focus reduction neutralization test (FFRNT) to measure of CCHFV-neutralizing antibodies.

**Results:**

Pseudo-plaque reduction neutralization test showed a high sensitivity (98%), specificity (100%) and agreement (96,6%) in qualitative comparison with those of the FFRNT. There was a high correlation between the titers obtained in PPRNT and FFRNT (R2 = 0.92). The inter- and intra-assay variation of PPRNT revealed good reproducibility and positive cut-off of PPRNT was defined as 1:4 by the geometric mean titers for the individual samples distributed.

**Conclusion:**

The pseudo-plaque reduction neutralization test described in this study is a fast, reproducible and sensitive method for the measurement of CCHF neutralizing antibodies. This novel assay could serve as useful tools for CCHF research in epidemiology, vaccine development and other studies of immunity. It also provides an alternative to PRNT when viruses with no or poor CPE in cell culture.

## Background

Crimean-Congo hemorrhagic fever virus (CCHFV) is a tick-borne virus of the genus *Nairovirus* family Bunyaviridae, which are enveloped viruses containing tripartite, negative polarity, single-stranded RNA [1,2]. Crimean-Congo hemorrhagic fever, a severe viral human disease, is characterized by sudden onset of fever, headache, abdominal pain, nausea, vomiting, extensive ecchymoses, bleeding, and hepatic dysfunction with fatality rates up to 30% [3,4]. The virus is transmitted to humans by the bite of infected ticks, by squashed ticks, or by exposure to the tissue or blood of infected livestock [5,6]. Crimean-Congo hemorrhagic fever virus can spread from person to person through contact with the tissue or blood of CCHF patients. It is one of the rare hemorrhagic fever viruses capable of inducing nosocomial outbreaks which may result in a more severe illness with a higher mortality rate [7-10].

Crimean-Congo hemorrhagic fever is diagnosed genetically by detection of viral RNA in acute-phase blood sample or serum [3,4,9-12]. Serological diagnosis relies on detection of anti-CCHF specific IgM and IgG in enzyme-linked immunosorbent (ELISA) and immunofluorescence assays (IFA) from paired acute and convalescent specimens [13-17]. Ideally, the confirmation of CCHF infection should be made by neutralization assay which is one of the most specific serological methods. Virus neutralization tests are usually based on the cytopathic effect (CPE) or the plaque-reduction neutralization test (PRNT) [18,19]. The CPE assay relies on the visual examination of the damage in magnified infected target cells. It is subjected to observer variation and it is difficult to make a quantitative determination of neutralizing activity based on the CPE. The PRNT has limitations for screening the large numbers of serum samples needed for epidemiological investigations. Neither CPE assay nor PRNT can be used to measure neutralization antibodies if the virus produces little or no CPE.

A pseudo-plaque reduction neutralization test (PPRNT) based on enzyme-catalyzed color development of infected cells probed with anti-CCHFV antibodies was used to measure neutralization antibody of CCHFV. The results obtained by PPRNT were compared with those of a fluorescence focus reduction neutralization test (FFRNT).

## Results

### CCHFV pseudo-plaque reduction neutralization assay

**Figure 1:**
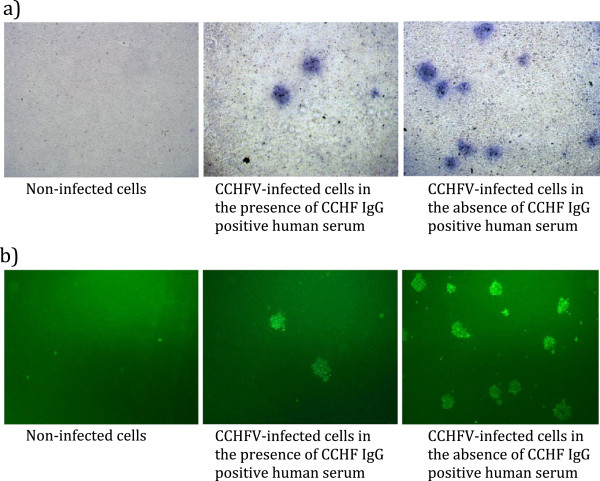
**Vero E6 cells infected with CCHFV Turkey-Kelkit06 under light microscopy (×40). (a)** and fluorescent microscopy (x40) **(b)** showing the presence (on the right) and absence (on the left) of pseudo-plaque forming units **(a)** and fluorescent focus-forming units **(b)**. Cells were stained 72 h post-infection (PI).

Crimean-Congo hemorrhagic fever Turkey-Kelkit06 strain does not produce plaques. We have been able to titrate the virus by the recently developed pseudo-plaque assay (PPA) described by Mitchell et al. [20] with some modifications. A pseudo-plaque reduction neutralization test was applied to CCHFV-neutralizing antibody detection in a 96-well microplate scale. Crimean-Congo hemorrhagic fever from challenged serial dilutions of human serum was grown on a Vero E6 cell line. After 3 days of infection and cell permeabilization, detection of the CCHFV pseudo-plaque was accomplished using polyclonal mouse anti-CCHFV serum primary antibody and β-gal-coupled anti-mouse IgG-antibody. The reaction was apparent with X-gal substrate. The viral pseudo-plaques stained medium blue to dark purple (Figure 1a). We also conducted a fluorescent focus reduction neutralization test (FFRNT) to measure of CCHFV-neutralizing antibodies to compare with PPRNT (Figure 1b).

### Reproducibility of pseudo-plaque reduction neutralization assay

In order to express reproducibility of the PPRNT results, we reported two measures of the Coefficient of Variability (CV); the Inter-Assay CV and the Intra-Assay CV. The CV is a number defined as the standart deviation of a distribution divided by its mean. In this study, the inter-assay CV expression of plate-to-plate consistency is calculated from the mean values for the high (n=2), medium (n=2) and low (n=2) controls on each plate. The six serum samples with known neutralization titers were tested by PPRNT and were run in duplicate in consecutive experiments to assess inter-assay variability (Table 1). To evaluate the intra-assay CV which is the average coefficient of variation between duplicates, sixty-nine human serum samples (20 acute and 49 convalescent) were tested five times by PPRNT (Table 1). The inter-assay coefficient of variation for samples ranged from 2.48% to 9.82% (mean 5.71%), whereas the intra-assay ranged from 4.63% to 10.81% (mean 7.44%)

**Table 1:** Intra and inter-assay variations in PPRNT

**Virus Input**	**Intra-assay variation**				**Inter-assay variation**		
	**Ratio between duplicate test in same serum**				*Six sera with known FFRNT		
	<4	4-7	7-10	Total	32 (n=2)	128 (n=2)	512 (n=2)
	n (%)	n (%)	n (%)	n	**GMT(CV)	GMT(CV)	GMT(CV)
50 PPFU/well	20 (28.98)	33 (47.85)	16 (23.18)	69	36.76 (4.63)	111.4 (7.70)	445.70 (10.81)
					27.86 (9.19)	147.0 (4.79)	588.10 (7.53)

*Neutralizing anti-CCHF antibodies titers were directly assigned to the highest dilution with > 50% reduction.

**GMT; geometric mean titer, CV; coefficient of variance = (S.D./mean) × 100%, which was calculated with logarithmic transformation.

### Comparison of PPRNT and FFRNT

**Figure 2:**
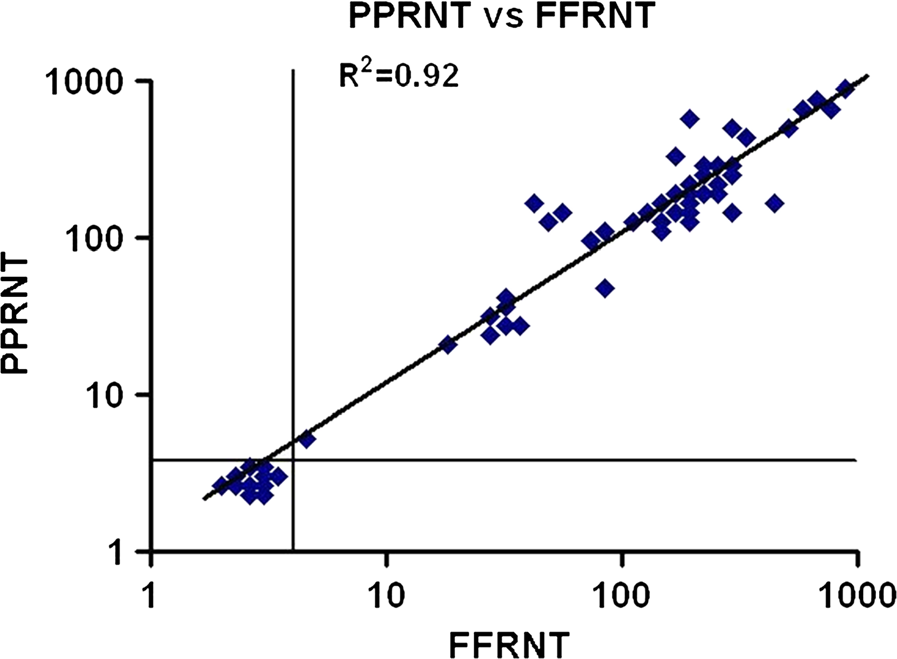
**Correlation of the mean neutralizing titers of 69 serum samples determined by FFRNT and PPRNT.** Based on the distribution, a positive cut-off value of 1:4 was defined for PPRNT and FFRNT. The solid line indicates complete correlation.

To validate to PPRNT for measuring CCHFV-neutralizing antibodies, 69 human sera (20 acute and 49 convalescent) with known FFRNT titers were tested by PPRNT. The results of FFRNT were regarded as the gold standard and as shown in Table 2, the sensitivity and specificity of PPRNT were 98 and 100%, respectively. The agreement and kapa statistics of PPRNT and FFRNT were 96.6 and 0.96%, respectively. A single sample of the 69 assessed was FFRNT positive, although it was negative by PPRNT (Table 2). From the distribution of geometric mean titers for the individual samples, a positive cut-off for FFRNT and PPRNT was defined as 1:4. There was a strong correlation between the titers obtained in PPRNT and FFRNT (R2 = 0.92; Figure 2).

**Table 2:** Qualitative comparison between PPRNT and FFRNT

	**FFRNT**		**Sensitivity (%)**	**Specificity (%)**	**Agreement (%)**	**Kappa**
	**> 4**	**< 4**	*(95% CI)	**(95% CI)**	**(95% CI)**	
PPRNT						
> 4	49	1	98.0	100	96.6	0.96
< 4	0	20				

*Fold differences were calculated based on the within and between assay standard deviation of log10 titres within which results from testing a sample twice should fall 95% of the time.

## Discussion

Crimean-Congo hemorrhagic fever virus is reported in many countries of Africa, Asia, the Middle East, and Eurasia [3,4], and the geographical distribution has expanded in recent decades. An increasing number of epidemics and sporadic cases have been reported in Kosovo, Bulgaria, Albania, Greece, the Russian Federation, Tajikistan, Kazakhstan, Georgia, Iran, Pakistan, Afghanistan, Mauritania, Senegal, Kenya, and India [9,10]. While serological evidence of CCHF has been found among humans since the 1970s, the first clinical CCHF infection was recognized in 2002. Currently, an exceptional outbreak of CCHF is occurring in Turkey with more than 7,000 cases reported creating a serious threat to public health [21-24].

Currently there are no specific treatments or licensed vaccines available for CCHFV. There have been few attempts to develop a vaccine because of the sporadic and limited numbers of cases, the lack of a suitable animal model to evaluate efficacy of vaccine candidates, and the high level biocontainment facilities required for working with the virus [25-27]. Recently, two research groups have found adult mice with defective interferon responses allowed to lethal CCHFV infection [28,29]. These mouse models could provide invaluable information for further studies. Efforts to develop a vaccine against CCHFV are being made.

To determine the efficacy of vaccine candidates it is important to conduct serological studies that can accurately measure levels of protective antibodies. Reports on measuring neutralizing antibodies against CCHFV are limited, and the neutralizing antibody response is weak and difficult to demonstrate in CCHF infections [26,30-32]. At present, neutralizing antibody titers to CCHF are most commonly measured using plaque reduction neutralization assay, which requires a monolayer of cells susceptible to the virus infection. However, the use of cell lines for CCHFV may produce little or no CPE, since the virus tends to develop a noncytopathic persistent infection, depending on the strain. An alternative method of measuring CCHF-neutralizing antibody response is fluorescent focus reduction neutralization assay (FFRNT). The two published reports on the detection of CCHF-neutralizing antibody with FFRNT are not recent [30,31]. In the present study, we developed PPRNT, based on colorimetric immunoassay used for infectious particle assay and clonal isolation of adeno-associated virus [20]. Fluorescent focus reduction neutralization assay was adapted for the measurement of neutralizing antibody responses against CCHF, and the results of FFRNT were regarded as the gold standard.

Parameters such as sensitivity, specificity, and reproducibility are important to evaluate when developing new or modified laboratory tests. In this study, the inter- and intra-assay variation of PPRNT revealed good reproducibility (Table 1). The PPRNT showed high sensitivity (98%), specificity (100%), and agreement (96.6%) in qualitative comparison with FFRNT (Table 2). The titers obtained by PPRNT and FFRNT were highly correlated (R2 = 0.92; Figure 2). In addition, we were able to define for both assays a positive cut-off (1:4) based on the geometric mean titers for the individual samples distributed. There was no significant difference between PPRNT and FFRNT with respect to determining CCHF-neutralizing antibody response. This is not surprising since both methods are based on the same fundamental principles and differ only in the surface area of the tissue culture plates and in the final visualization steps. However, a unique advantage of PPRNT is that the pseudo-plaques can be counted using the naked eye or by light microscopy. Moreover, the plates containing pseudo-plaques can be stored for an extended period of time.

Unlike the standard plaque reduction neutralization test, PPRNT does not require cell destruction or damage by the virus infection. It allows the use of virus strains that do not form plaques, such as CCHFV Turkey-Kelkit06, for the measurement of neutralizing antibodies. Therefore, results can be more rapidly obtained by PPRNT (3 days after infection) than with PRNT (usually 5 to 7 days after infection). Another potential advantage of the PPRNT compared to standard PRNT is that PPRNT is performed in 96-well plates, enabling investigation of a large number of samples.

In conclusion, the pseudo-plaque neutralization assay described here is a rapid, reproducible, and sensitive method for the measurement of CCHF neutralizing antibodies. This novel, high-throughput assay could serve as a useful tool for CCHF research in epidemiology, vaccine development, and other studies of immunity. It also provides an alternative to PRNT when viruses with no or poor CPE in cell culture.

## Methods

### Cells, virus, and antibodies

Vero E6 cells (African green monkey kidney) obtained from ATCC (CRL 1586) were maintained in Dulbecco’s modified Eagle’s medium (DMEM) supplemented with 10% heat-inactivated fetal bovine serum (FBS), 100 mM Lglutamine, 50 U ml^-1^ penicillin, and 50 μg ml^-1^ streptomycin (Sigma-Aldrich, Germany).

The CCHFV Turkey-Kelkit06 strain was isolated from a patient in Turkey [33]. CCHFV Turkey-Kelkit06 was passaged three times by intracerebral inoculations of 2-3 day old suckling mice. The CCHFV stocks were prepared on Vero E6 cells by infection of T175 cell culture flasks with a 1:100 dilution. Supernatants were collected on days 4 and 5 post-infection (PI), cleared of cell debris by centrifugation at 500 g for 20 min at 4°C, and aliquots were stored at -80°C. The titer of the viral stock was quantified at 4.3×10^5^ ml^-1^ using a pseudo-plaque assay [34]. All handling of virus was conducted in a biosafety level 3 laboratory (BSL-3).

To produce the primary antibodies, after cultivation of CCHFV infected Vero E6 cells the viral particles were obtained by using sucrose gradient ultracentrifugation and the gradients which intensely contain viral specific particles were collected to make purification of the virus. Inactivation of the virus by formaldehyde was followed by dialysis (MWCO 20 kDa, Millipore, Bedford, MA) with phosphate-buffered saline. The protein concentration of the fractions were determined a colorimetric assay (Bicinchoninic acid protein assay kit, Sigma–Aldrich). New Zealand white rabbits received 100 microgram of the viral antigen adjuvanted with ALUM on day 1, 21, 42. Balb-C mice received 50 microgram of the viral antigen adjuvanted with ALUM on day 1, 21, 42. Blood samples were taken every week and tested by ELISA to determine whether they`re positive anti-CCHFV IgG. After purification of the IgGs from the rabbits and mice they were used as primary antibodies in this method.

The entire protocol and the animal experiments were approved by the Ethics Committee of Firat University**.**

### Serum samples

Sixty-nine human serum samples (20 acute and 49 convalescent) were tested. The presence of CCHFV RNA was confirmed by RT-PCR for the acut samples as described elsewhere [35]. The presence of anti-CCHFV IgG for the 49 convalescent was confirmed by a commercial ELISA kit (vectorbest®Russia). Written informed consent for participation in the study was obtained from participants. All sera were heated at 56°C for 30 min to eliminate serum complement.

### Pseudo-plaque reduction neutralization test (PPRNT)

The CCHFV pseudo-plaque reduction neutralization assay was performed in 96-well microtiter plates. Vero E6 cells were grown to confluence with DMEM containing 10% FBS in 96-well micro titer plate (Corning, USA) at 37°C, 5% CO_2_ for 18-24 h. Human serum samples diluted 1:4 in Eagle’s MEM (EMEM) were heat-inactivated at 56°C for 30 min. Two-fold serial dilutions from 1:4 to 1:4.096 were prepared in virus diluent (EMEM with L-glutamine containing 1% FBS, 2% HEPES (1 M), and 1% penicillin, streptomycin 100×). Serial dilutions of the test specimens were challenged with approximately 50 pseudo-plaque forming units (ppfu) incubated overnight at 4°C. Two hundred microliters of the serum–virus mixtures were adsorbed to confluent cell monolayers (in duplicate) and incubated for an additional hour at 37°C. The supernantant was removed, and the cell monolayer was overlaid with the virus medium (DMEM with L-glutamine containing 2% FBS, 2% HEPES (1 M), and 1% penicillin, streptomycin 100×) supplemented with 1% carboxy methyl cellulose (Sigma-Aldrich, Germany) and incubated at 37°C, 5% CO_2_ for 3 days. The cells were fixed with a solution of 10% buffered neutral formaldehyde in PBS. After 20 min the formaldehyde solution was aspirated, the cells were washed twice with TBST (100 mM Tris-HCl pH 8.0, 1.5 M NaCl, 1% Tween 20), permeated with 0.1% Triton X 100 in PBS for 20 min and blocked with 5% skim milk in PBS. Polyclonal mouse anti-CCHFV serum (1:1500) was added to each well in TBST and incubated for 1 h at room temperature with gentle rocking. Following three 10 min washes in TBST, goat anti-mouse β-gal conjugate diluted 1:1500 in TBST (Southern Biotech, USA) was added to each well and incubated 1 h at room temperature with gentle rocking. The cells were washed five times with TBST, and the substrates nitro blue tetrazolium (NBT) and X-gal (5-bromo-4-choloro-3-indolyl-beta-D-galactopyranoside) were added to each well and incubated at 37°C. Microplates were checked microscopically every 10 min. Neutralizing anti-CCHF antibody titers were directly assigned to the highest dilution with > 50% reduction.

### Fluorescent focus reduction neutralization test (FFRNT)

Vero E6 cells at density of 3×10^5^ ml^-1^ were seeded into Lab Tek II 8-well chamber slides (Sigma-Aldrich, Germany) at 37°C and 5% CO2 to achieve 80-90% confluence the following day. Sera, including positive and negative controls, were diluted 1:4 in EMEM inactivated at 56°C for 30 min and serially diluted two-fold in EMEM in 96-well tissue culture microplates. The plates were incubated at 4°C overnight with an equal volume CCHFV Turkey-Kelkit06 to provide approximately 50 ppfu per 100 μl. Two hundred microliters of the serum–virus mixtures were added in duplicate to cell monolayers and allowed to adsorb for 1 h at 37°C. Inoculums were aspirated, and slides were overlaid with 1% carboxy methyl cellulose (Sigma-Aldrich,Germany) and incubated at 37°C and 5% CO_2_ for 3 days. After fixation with 10% buffered neutral formaldehyde, the cells were permeated with 0.1% Triton X 100 in PBS for 20 min and blocked with 5% skim milk in PBS. Slides were incubated with rabbit anti-CCHFV polyclonal antisera (1:1000) for 1 h in TBST at 37°C in a humid chamber. Following three 10 min washes in TBST, antibody-labeled cells were detected by incubation for 1 h with goat anti-rabbit IgG conjugated with fluorescein isothiocyanate (FITCH) (Southern Biotech, USA) and diluted 1:1000 in TBST. After extensive washing, the cells were mounted in anti-fading medium (Sigma-Aldrich,Germany) and analyzed by immunofluorescence microscopy (Olympus BX50, Japan). Neutralizing anti-CCHF antibodies titers were directly assigned to the highest dilution with > 50% reduction.

### Statistical analysis

Statistical analysis was carried out using Graphpad Prism 5. The 95% confidence interval (95% CI) of sensitivity and specificity were calculated. The intra-assay and inter-assay variations of the neutralization test (NT) results were determined and the result was given as a coefficient of variation (CV) and geometric mean titer (GM). Agreement and Kappa values were calculated using SPSS 17.

## Competing interests

The authors declare that they have no competing interests.

## Authors' contributions

NC and EB carried out the study. MDY, ST, and YB participated in the immunoassays. MA, AK and ME participated in the design of the study and performed the statistical analysis. AO conceived of the study, and participated in its design and coordination and helped to draft the manuscript. All authors read and approved the final manuscript.
